# Hydrophobic Ti_3_C_2_T_x_/TEMPO Oxidized Cellulose Nanofibers Composite Aerogel for Efficient Oil-Water Separation

**DOI:** 10.3390/polym17030273

**Published:** 2025-01-22

**Authors:** Yaqing Guo, Juncheng Zhang, Siyuan Wang, Xiyue Li, Yuanyuan Miao, Jing Zhou, Zhenbo Liu

**Affiliations:** Key Laboratory of Bio-Based Material Science & Technology of Ministry of Education, Northeast Forestry University, Harbin 150040, China; guoyaqing0209@163.com (Y.G.); wangsiyuan202412@163.com (S.W.); y17703327767@163.com (X.L.); miao.yuanyuan@nefu.edu.cn (Y.M.); zj@nefu.edu.cn (J.Z.)

**Keywords:** TEMPO oxidized cellulose nanofibers, Ti_3_C_2_T_x_, aerogel, hydrophobic, oil-water separation

## Abstract

To address the pollution issues of industrial oily wastewater and catering industry wastewater, a series of Ti_3_C_2_T_x_/TEMPO oxidized cellulose nanofibers composite aerogels with varying Ti_3_C_2_T_x_ content were successfully prepared using liquid nitrogen non-directional and directional freezing methods, with Ti_3_C_2_T_x_ and TEMPO oxidized cellulose nanofibers (TOCNF) as the main raw materials. The prepared samples were then hydrophobically modified using methyltrichlorosilane (MTCS) via chemical vapor deposition (CVD). The results showed that the directional Ti_3_C_2_T_x_/TOCNF composite aerogel had the most orderly SEM morphology. The hydrophobic Ti_3_C_2_T_x_/TOCNF composite aerogels exhibited efficient adsorption separation capabilities, with an adsorption capacity ranging from 21.5 to 78.2 times their own mass. Notably, the oil absorption performance was optimal when the mass fraction of Ti_3_C_2_T_x_ was 33.3%. After five adsorption cycles, the adsorption capacity of M5C10 (with a mass ratio of Ti_3_C_2_T_x_ to TOCNF of 5:10) only decreased by around 11%. M5C10 exhibits highly efficient oil absorption performance, which is of considerable significance for the research on oil-water separation treatment of industrial wastewater, domestic wastewater, and sewage from the catering industry.

## 1. Introduction

Oil pollution in water has become a significant issue that poses a threat to human health and environmental safety due to the continuous and large-scale discharge of oily wastewater from industrial production processes and pollution caused by the catering industry [1,2,3]. Therefore, there is an urgent need to develop simple, effective, and economical methods to treat oily wastewater. Compared to chemical and biological approaches, physical treatment approaches possess the advantages of being convenient to operate, highly efficient, and free from secondary pollution when implemented on a large scale [4,5]. Currently, a large number of researchers have carried out profound investigations into a diverse range of oil adsorption materials [6]. Among them, materials with a three-dimensional structure, low density, and high porosity are favored in oil-water separation [7]. For example, foams [8], expandable graphite [9], aerogels [10], and sponges [11] are some of the materials being explored. In contrast, aerogels are readily available, have a minimal environmental impact, and exhibit superior reusability and efficiency in oil-water separation [12,13,14].

Biodegradable, readily available, cost-effective, and environmentally friendly biomass aerogels are attracting increasing attention [15]. Biomass aerogels are primarily derived from various types of bio-based materials such as cellulose [16] and serve as substrates in oil-water separation processes [5,17]. Cellulose is derived from biodegradable resources such as wood. It can be broken down naturally, imposing no burden on the environment and causing no pollution. Cellulose-based aerogels not only have high porosity, low density, and high specific surface area but also possess excellent adsorption capabilities and degradability [18,19,20,21]. However, the presence of a large number of hydroxyl groups in cellulose molecules endows them with strong water absorption [22]. When cellulose aerogel materials are directly used to treat oily wastewater, they absorb a significant amount of water while adsorbing oil, leading to poor oil-water selectivity and low separation efficiency, thus affecting their effectiveness [23,24]. Ti_3_C_2_T_x_, a new class of two-dimensional materials, is considered a promising material for oil-water separation due to its high specific surface area [25,26].

Aerogels possess a relatively high porosity, which provides a large number of active sites for the adsorption of oil molecules [27,28]. Their low density enables them to float effortlessly on the water surface, ensuring close contact with floating oil [29]. In addition, through fluorination, silanization, and the introduction of flexible nanomaterials, aerogels can be made highly hydrophobic and oleophilic. This property dictates that when aerogels come into contact with an oil-water mixture, oil droplets are rapidly attracted and adsorbed while water is repelled, facilitating efficient oil-water separation. Lu et al. [30] prepared graphene/cellulose aerogels with highly efficient solar-driven crude oil purification. Rejeb et al. [31] prepared hydrophobic ambient-dried molecularly-bridged silica aerogels with efficient and versatile oil/water separation. Wang et al. [32] prepared hydrophobic polyimide/Ti_3_C_2_T_x_ aerogels that are interconnected, highly porous, and of low density. Therefore, the development of aerogels with durability, degradability, and excellent oil absorption performance through a simple process has become a major research focus.

Herein, to address the issue of oil-based water pollution and develop a more efficient aerogel for oil-water separation, TEMPO oxidized cellulose nanofibers (TOCNF) are used as the skeleton, with Ti_3_C_2_T_x_ as the reinforcing phase and methyltrichlorosilane (MTCS) as the hydrophobic modification agent to prepare hydrophobic Ti_3_C_2_T_x_/TOCNF composite aerogels with varying Ti_3_C_2_T_x_ content. With the anticipation of developing an oil-absorbing material possessing high oil-absorption capacity and efficient oil-water separation capability, the adsorption effects for oil-water separation will be investigated, which aims to pave new ways for the efficient and clean resource utilization of oily wastewater.

## 2. Materials and Methods

### 2.1. Materials

TEMPO oxidized cellulose nanofibers (TOCNF) were purchased from Tianjin Woodelf Biotechnology Co., Ltd. (Tianjin, China). Hydrochloric acid (HCl, AR) was purchased from Tianjin Comio Chemical Reagents Co., Ltd. (Tianjin, China). Titanium aluminum carbide (Ti_3_AlC_2_), hydrofluoric acid (HF), lithium chloride (LiCl), and methyltrichlorosilane (MTCS) were purchased from Shanghai Yien Chemical Technology Co., Ltd. (Shanghai, China). Deionized water was prepared in-house in the laboratory. All chemicals were utilized as received, without further purification.

### 2.2. Preparation of Ti_3_C_2_T_x_

In a polytetrafluoroethylene (PTFE) container, 18 mL of H_2_O was first added. Then, under continuous magnetic stirring and strict temperature control, 36 mL of 12 mol/L HCl was slowly added to the H_2_O. After that, 6 mL of 40% HF was added dropwise and the mixture was magnetically stirred for 10 min to ensure uniform mixing. Then, 2 g of Ti_3_AlC_2_ was gradually and slowly added to the container in small portions over approximately 3 min, and the mixture was continuously stirred at 35 °C for 24 h. Subsequently, the mixture was centrifuged at 5000 rpm for 5 min, and this process was repeated until the pH value of the supernatant in the centrifuge tube reached 7, after which the supernatant was discarded. 1 g of LiCl was dissolved in 50 mL of deionized water, and the aforementioned precipitate was added to it and stirred at room temperature for 8 h. The mixture was then centrifuged at 3500 rpm for 5 min, and this operation was repeated until the supernatant turned black. The mixture was further shaken by hand until the liquid in the centrifuge tube became viscous and thick. Finally, the Ti_3_C_2_T_x_ dispersion was centrifuged at 3500 rpm for 30 min, and the upper liquid was collected to obtain a single-layer Ti_3_C_2_T_x_ dispersion. The dispersion was then frozen with liquid nitrogen and placed into a vacuum freeze dryer for 48 h to yield Ti_3_C_2_T_x_ powder.

### 2.3. Preparation of Hydrophobic Ti_3_C_2_T_x_/TOCNF Composite Aerogels

TOCNF and Ti_3_C_2_T_x_ powders were mixed in mass ratios of 10:1, 5:1, 2:1, 1:1, and 1:2 to obtain five different Ti_3_C_2_T_x_/TOCNF suspensions. These suspensions were placed on a magnetic stirrer and stirred uniformly at 600 rpm for 5 h to form a homogeneous and stable suspension. The suspensions were then poured into molds and completely frozen using both directional and non-directional liquid nitrogen freezing methods. The frozen samples were placed into a vacuum freeze dryer for 48 h to obtain the Ti_3_C_2_T_x_/TOCNF composite aerogels.

The Ti_3_C_2_T_x_/TOCNF composite aerogels were subjected to hydrophobic modification via the chemical vapor deposition (CVD) method. The aerogels were placed in a beaker containing MTCS, sealed, and left at room temperature for 24 h. After the treatment, the aerogels were stored in an oven at 60 °C for 24 h to remove excess saltwater, resulting in hydrophobic aerogels.

### 2.4. Performance Testing and Characterization

The microstructure of the composite aerogel was observed using a scanning electron microscope (SEM, Apreo S HiVac, Thermo Scientific, Waltham, MA, USA). The chemical structure of the composite aerogel was analyzed using a Fourier-transform infrared spectrometer (FT-IR, Nicolet iN10, Thermo Scientific, Waltham, MA, USA) with a test range of 500 to 4000 cm^−1^. The phase structure of the composite aerogel was analyzed using an X-ray diffractometer (XRD, SmartLab 9KW, Rigaku, Tokyo, Japan) with settings of 2θ from 5° to 90° at a scanning speed of 10 °/min. The thermal stability of the composite aerogel was analyzed using a thermogravimetric analyzer (TG, TG 209 F1, NETZSCH, Selb, Germany) with a temperature range set from 30 to 800 °C. The hydrophobicity of the composite aerogel was analyzed using a contact angle meter (OCA20, Dataphysics, Filderstadt, Germany).

Ethanol, soybean oil, vacuum pump oil, and n-hexane were selected to study the adsorption performance of the composite aerogel. Each sample was cut into three rectangular pieces with dimensions of approximately 2 cm × 1 cm × 1 cm. The initial mass of the samples was measured. Then, 30 mL of ethanol, soybean oil, vacuum pump oil, and n-hexane were each added to three 50 mL beakers, respectively. The samples were immersed and soaked for 30 min to reach adsorption equilibrium. Afterward, the samples were taken out, and excess organic solvents around the samples were blotted with filter paper. The samples were then weighed again, and the results were taken as the average of three measurements. The oil absorption rate was calculated using the following formula:

(1) $$Q=\frac{M_{1}-M_{0}}{M_{0}}\times 100\%$$

Here, *Q* represents the oil absorption rate; *M*_1_ represents the mass of the sample after oil absorption; *M*_0_ represents the mass of the sample before oil absorption.

The reusability performance of the composite aerogel was analyzed. Desorption experiments and adsorption cycles were conducted. The samples, after being saturated with oil, were subjected to oil extraction, followed by repeated rinsing with anhydrous ethanol and squeezing. This process was repeated three times before the samples were placed in an oven at 60 °C for 8 h to dry. The desorbed samples were then retested for oil-water separation. The aforementioned operations were repeated four times, and the oil adsorption capacity of the composite aerogel was recorded each time.

## 3. Results

### 3.1. The Microstructure of Ti_3_C_2_T_x_

**Figure 1:**
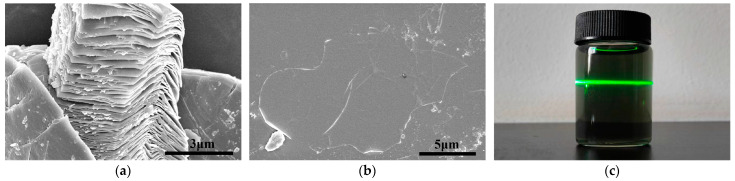
(**a**) SEM image of multi-layer Ti_3_C_2_T_x_; (**b**) SEM image of single-layer Ti_3_C_2_T_x_; (**c**) The Tyndall effect of Ti_3_C_2_T_x_ dispersion liquid.

By etching the closely packed precursor Ti_3_AlC_2_ with a mixed solution of HCl/HF, accordion-like multilayered Ti_3_C_2_T_x_ was obtained, as shown in Figure 1a. Further intercalation treatment of the multilayered Ti_3_C_2_T_x_ was carried out using Li^+^ in a LiCl solution. Manual shaking and centrifugation were employed to exfoliate it into two-dimensional single-layer Ti_3_C_2_T_x_. As depicted in Figure 1b, this indicates successful etching and exfoliation of the material. As observed in Figure 1c, the single-layer Ti_3_C_2_T_x_ dispersion exhibits a uniform dark green color and a distinct Tyndall effect, which indicates the excellent dispersibility of Ti_3_C_2_T_x_ in aqueous solutions.

### 3.2. The Morphological Analysis of Ti_3_C_2_T_x_/TOCNF Composite Aerogels

**Figure 2:**
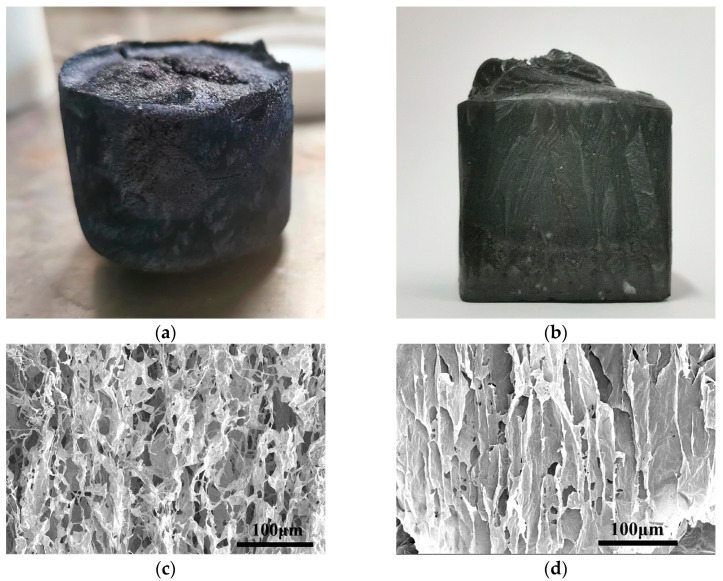
(**a**) The appearance and morphology of non-directional Ti_3_C_2_T_x_/TOCNF composite aerogels; (**b**) The appearance and morphology of directional Ti_3_C_2_T_x_/TOCNF composite aerogels; (**c**) SEM images of non-directional Ti_3_C_2_T_x_/TOCNF composite aerogels; (**d**) SEM images of directional Ti_3_C_2_T_x_/TOCNF composite aerogels.

The macroscopic morphology of non-directional and directional Ti_3_C_2_T_x_/TOCNF composite aerogels (Figure 2a,b) appeared relatively uniform, with a slightly white surface. This was due to a small amount of TOCNF being carried to the upper surface of the aerogel during the rapid freezing process. However, the Ti_3_C_2_T_x_ dispersion and TOCNF were well combined, showing a stable state. The non-directional Ti_3_C_2_T_x_/TOCNF composite aerogel had a denser structure of pores, but the pores were relatively disordered (Figure 2c). In contrast, the directional freezing technology contributed to the directional growth of internal crystals in the directional Ti_3_C_2_T_x_/TOCNF composite aerogel (Figure 2d). Therefore, a directional liquid nitrogen freeze-drying process was adopted to prepare composite aerogels with uniform macroscopic morphology and orderly microstructure.

**Figure 3:**
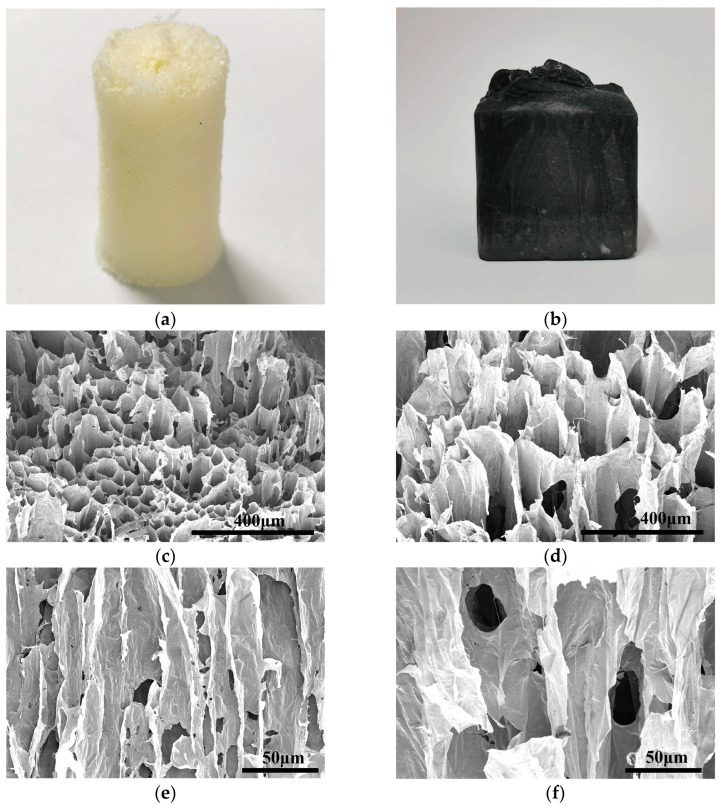
(**a**) The appearance and morphology of TOCNF aerogel; (**b**) The appearance and morphology of hydrophobic Ti_3_C_2_T_x_/TOCNF composite aerogel; (**c**) SEM images of the cross-section of TOCNF aerogel; (**d**) SEM images of the cross-section of hydrophobic Ti_3_C_2_T_x_/TOCNF composite aerogel; (**e**) SEM images of the vertical section of TOCNF aerogel; (**f**) SEM images of the vertical section of hydrophobic Ti_3_C_2_T_x_/TOCNF composite aerogel.

The TOCNF aerogel appeared white (Figure 3a). The hydrophobic Ti_3_C_2_T_x_/TOCNF composite aerogel was black (Figure 3b), with a complete appearance and stable structure. However, the pure Ti_3_C_2_T_x_ aerogel was brittle and difficult to form a stable structure. For the hydrophobic Ti_3_C_2_T_x_/TOCNF composite aerogel, the surface of TOCNF is rich in active functional groups, such as —COOH and —OH, which is comparable to the surface characteristics of Ti_3_C_2_T_x_, which contains active groups such as —OH, —O, and —F. Consequently, there is a significant interfacial interaction between TOCNF and Ti_3_C_2_T_x_ due to hydrogen bonding between the surface-active groups of both materials [33]. Under the action of hydrogen bonds, TOCNF and Ti_3_C_2_T_x_ bind tightly, forming a more orderly network. Compared to the pure TOCNF aerogel (Figure 3e), the hydrophobic Ti_3_C_2_T_x_/TOCNF composite aerogel (Figure 3f) had fewer pores, with the pores being filled between each other, possessing an orderly arranged directional porous structure. Due to the directional freezing process [34], ice crystals grew rapidly from the bottom to the top under the influence of temperature differences, resulting in a more orderly and closely connected porous structure (Figure 3c,d).

### 3.3. The Chemical Structure Analysis of Ti_3_C_2_T_x_/TOCNF Composite Aerogels

**Figure 4:**
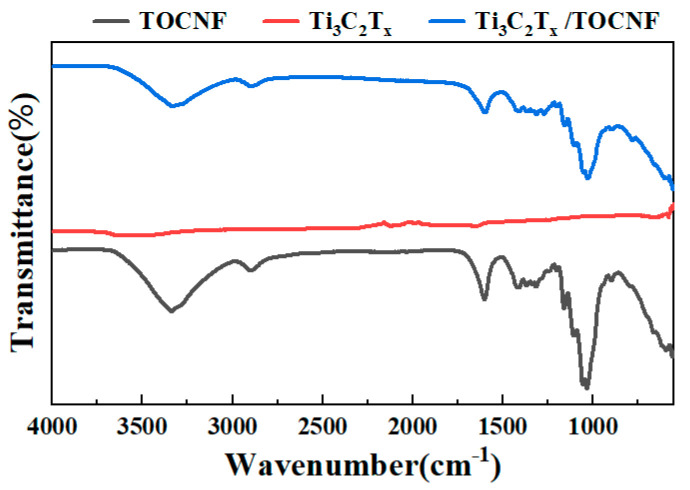
FT-IR spectra of TOCNF, Ti_3_C_2_T_x,_ and hydrophobic Ti_3_C_2_T_x_/TOCNF composite aerogels.

The FTIR spectra (Figure 4) exhibit several characteristic peak bands of the TOCNF aerogel at 3369 cm^−1^, 2899 cm^−1^, and 1034 cm^−1^, which are attributed to the stretching vibrations of —OH, C—H, and C—O bonds, respectively [35]. Two absorption peaks of Ti_3_C_2_T_x_ at 1649 cm^−1^ and 3449 cm^−1^ are owing to the stretching vibrations of C=O and —OH, confirming the presence of oxygen-containing functional groups on the surface of Ti_3_C_2_T_x_ [36]. Compared to the TOCNF aerogel, the C—O stretching vibration peak of the hydrophobic Ti_3_C_2_T_x_/TOCNF composite aerogels exhibits a shift towards a smaller wavenumber, specifically from 1034 cm^−1^ to 1027 cm^−1^. Similarly, the —OH stretching vibration peak of the hydrophobic Ti_3_C_2_T_x_/TOCNF composite aerogels also indicates a shift towards a smaller wavenumber, exactly from 3369 cm^−1^ to 3350 cm^−1^. The stretching vibration peak of the C=O bond shifted from 1633 cm^−1^ to 1625 cm^−1^. These shifts indicate a strong hydrogen bond interaction between TOCNF and Ti_3_C_2_T_x_, which promotes a better formation of the nanostructures of Ti_3_C_2_T_x_ and TOCNF [37]. After the introduction of MTCS into the composite aerogel, the peak position of —OH remains constant [38]. Additionally, two other absorption peaks were observed at 781 cm^−1^ and 1271 cm^−1^, which are attributed to the stretching vibration of the Si—C single bond and the deformation vibration of —CH_3_, respectively [39]. New characteristic peaks appeared at 1615 cm^−1^ and around 578 cm^−1^ in the hydrophobic Ti_3_C_2_T_x_/TOCNF composite aerogels. In contrast to the TOCNF aerogel, these peaks correspond to the C=O bond and the Ti—O bond [40] of Ti_3_C_2_T_x_. This is due to the addition of Ti_3_C_2_T_x_, indicating that Ti_3_C_2_T_x_ has been successfully combined with the TOCNF to obtain the composite aerogel.

### 3.4. The Phase Structure Analysis of Ti_3_C_2_T_x_/TOCNF Composite Aerogels

**Figure 5:**
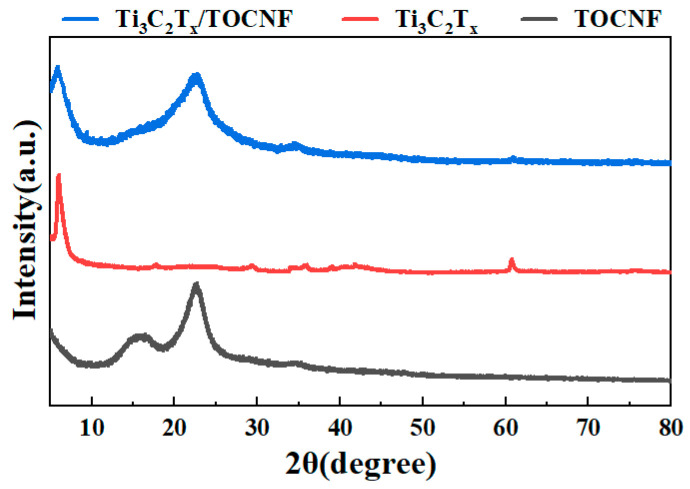
XRD spectra of TOCNF, Ti_3_C_2_T_x_, and hydrophobic Ti_3_C_2_T_x_/TOCNF composite aerogels.

As observed in the XRD patterns (Figure 5), there is a prominent peak at 2θ = 6.6°, corresponding to the (002) peak of Ti_3_C_2_T_x_ [41]. The characteristic peak (2θ = 6.0°) in the spectrum of the hydrophobic Ti_3_C_2_T_x_/TOCNF composite aerogel shifts to the left, indicating that the combination of TOCNF and Ti_3_C_2_T_x_ reduces the interlayer spacing of Ti_3_C_2_T_x_. The characteristic peaks of the TOCNF aerogels are located near 15° and 22° in the X-ray diffraction pattern [42,43,44], while the characteristic peaks of Ti_3_C_2_T_x_ appear approximately at 6.6° and 60° in the crystal plane diffraction peaks. Therefore, it can be seen that the hydrophobic Ti_3_C_2_T_x_/TOCNF composite aerogels not only contain the characteristic diffraction peaks of the TOCNF aerogel but also exhibit the characteristic peaks of Ti_3_C_2_T_x_. This is in agreement with the infrared results. It confirms that Ti_3_C_2_T_x_ has been successfully doped into the cellulose and indicates the successful preparation of the composite aerogel.

### 3.5. The Thermal Stability Analysis of Ti_3_C_2_T_x_/TOCNF Composite Aerogels

**Figure 6:**
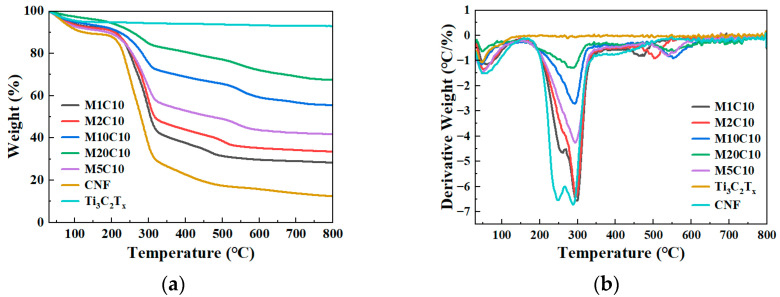
(**a**) TG curves of TOCNF, Ti_3_C_2_T_x_, and hydrophobic Ti_3_C_2_T_x_/TOCNF composite aerogels with different Ti_3_C_2_T_x_ mass fractions; (**b**) DTG curves of TOCNF, Ti_3_C_2_T_x_, and hydrophobic Ti_3_C_2_T_x_/TOCNF composite aerogels with different Ti_3_C_2_T_x_ mass fractions.

From the TG and DTG curves (Figure 6), it can be observed that before the temperature rises to 114 °C, all samples experienced a slight mass loss, which is likely due to the evaporation of inherent moisture. The structural changes in this stage were relatively simple, mainly involved the removal of water, and had little impact on the overall chemical structure of the samples. Ti_3_C_2_T_x_ did not exhibit any mass gain during the heating process, and the total mass loss was only about 7.13%, which indicated that Ti_3_C_2_T_x_ had high thermal stability. When the temperature reached 205 °C, TOCNF and hydrophobic Ti_3_C_2_T_x_/TOCNF composite aerogels with different Ti_3_C_2_T_x_ mass fractions began to undergo a second weight loss. At this stage, rapid thermal decomposition removed hydrogen and oxygen, led to the rearrangement of free carbon atoms, triggered intramolecular cyclization and intermolecular aromatization reactions, and ultimately condensed into a microcrystalline carbon structure. During this stage, significant changes took place in the chemical structure of the aerogel. The initial degradation temperature (T_0_) of the hydrophobic Ti_3_C_2_T_x_/TOCNF composite aerogels was 184 °C, and the maximum degradation temperature (T_max_) was 327 °C. The results indicated that the hydrophobic Ti_3_C_2_T_x_/TOCNF composite aerogels possessed outstanding thermal stability, and this thermal stability increased with the addition of more Ti_3_C_2_T_x_ mass.

### 3.6. The Water Contact Angle Analysis of Ti_3_C_2_T_x_/TOCNF Composite Aerogels

**Figure 7:**
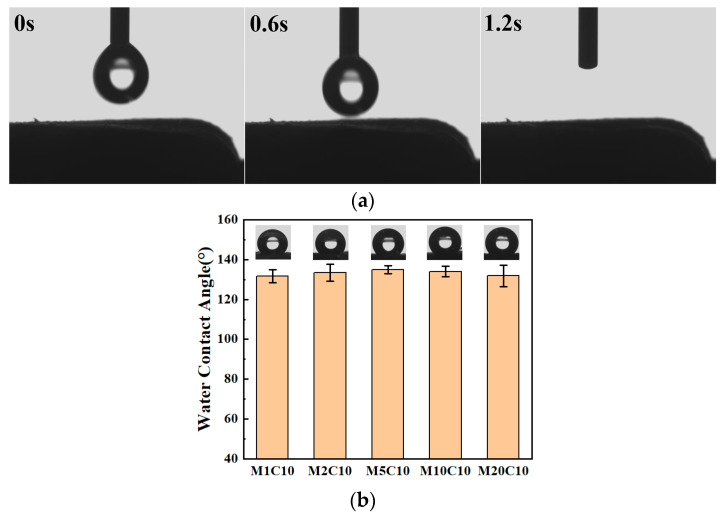
(**a**) Water contact angle of Ti_3_C_2_T_x_/TOCNF composite aerogel; (**b**) Water contact angle of hydrophobic Ti_3_C_2_T_x_/TOCNF composite aerogels with different Ti_3_C_2_T_x_ mass fractions.

Unmodified Ti_3_C_2_T_x_/TOCNF composite aerogels exhibited strong hydrophilicity, with water droplets being absorbed within 0.6 s (Figure 7a). This characteristic was attributed to their main raw materials—both Ti_3_C_2_T_x_ and TOCNF were hydrophilic materials. The surface of Ti_3_C_2_T_x_ carried hydrophilic groups —O/—OH [45], and TOCNF was rich in —OH [44]. These groups could form a large number of hydrogen bonds with water molecules, thus endowing the Ti_3_C_2_T_x_/TOCNF composite aerogels with hydrophilicity. However, as shown in Figure 7b, after hydrophobic modification, Ti_3_C_2_T_x_/TOCNF composite aerogels with different Ti_3_C_2_T_x_ mass fractions exhibit water contact angles all greater than 130°, demonstrating significant hydrophobicity. The main reason for this transformation was that during the chemical vapor deposition process, MTCS reacted chemically with Ti_3_C_2_T_x_ and TOCNF, effectively eliminating the original hydrophilic groups. Furthermore, as the content of Ti_3_C_2_T_x_ increased, the micro-nano structure of the hydrophobic Ti_3_C_2_T_x_/TOCNF composite aerogels became more complex and richer, which further enhanced the hydrophobicity of the hydrophobic Ti_3_C_2_T_x_/TOCNF composite aerogels.

### 3.7. The Application of Hydrophobic Ti_3_C_2_T_x_/TOCNF Composite Aerogels in Oil-Water Separation

#### 3.7.1. Adsorption Capacity Analysis

**Figure 8:**
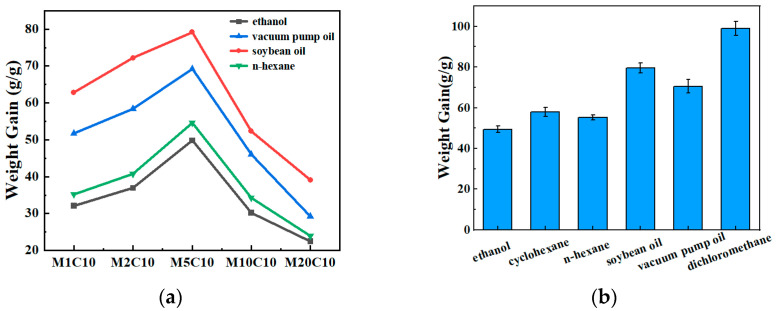
(**a**) Adsorption capacity of hydrophobic Ti_3_C_2_T_x_/TOCNF composite aerogel for ethanol, soybean oil, vacuum pump oil, and n-hexane; (**b**) Adsorption capacity of M5C10 for different types of oils and organic solvents.

Figure 8a visually illustrates the adsorption capacities of hydrophobic Ti_3_C_2_T_x_/TOCNF composite aerogels with varying Ti_3_C_2_T_x_ mass fractions for ethanol, soybean oil, and vacuum pump oil. The data from the figure indicate that these composite aerogels exhibit excellent adsorption capabilities, with adsorption amounts ranging from 21.5 to 78.2 times their own mass. Among them, the M5C10 composite aerogel demonstrates the highest adsorption capacity among these samples, suggesting that the microporous structure of the hydrophobic Ti_3_C_2_T_x_/TOCNF composite aerogels effectively adsorbs organic solvent oils. However, when a certain amount of Ti_3_C_2_T_x_ was added to the aerogel, the porosity of the hydrophobic Ti_3_C_2_T_x_/TOCNF composite aerogel decreased, which consequently led to a reduction in its adsorption capacity. When exceeding a certain limit, the hydrophobic capability decreases. When the content of Ti_3_C_2_T_x_ increases, its absorption capacity decreases, which may be related to the decrease in specific surface area or pore volume [38].

To quantitatively measure its adsorption capacity, M5C10 was selected to adsorb ethanol, cyclohexane, n-hexane, soybean oil, vacuum pump oil, and dichloromethane (Figure 8b). It was found that M5C10 exhibited a very high adsorption capacity for all the above-mentioned solvents. This is mainly because M5C10 has a large specific surface area and abundant pores. Meanwhile, its excellent adsorption performance makes it an ideal choice for removing pollutants such as oils and organic solvents. Notably, M5C10 exhibited particularly outstanding adsorption capacity for dichloromethane. The maximum adsorption capacity was as high as 98.92 g/g, which was higher than some adsorbent materials reported in the past (Table 1). The M5C10 composite aerogel has a low density, good hydrophobicity, and excellent adsorption capacity, along with other advantages. Therefore, it shows broad application prospects in the treatment of industrial oily wastewater and catering industry wastewater.

#### 3.7.2. Selective Oil Adsorption Analysis

**Figure 9:**
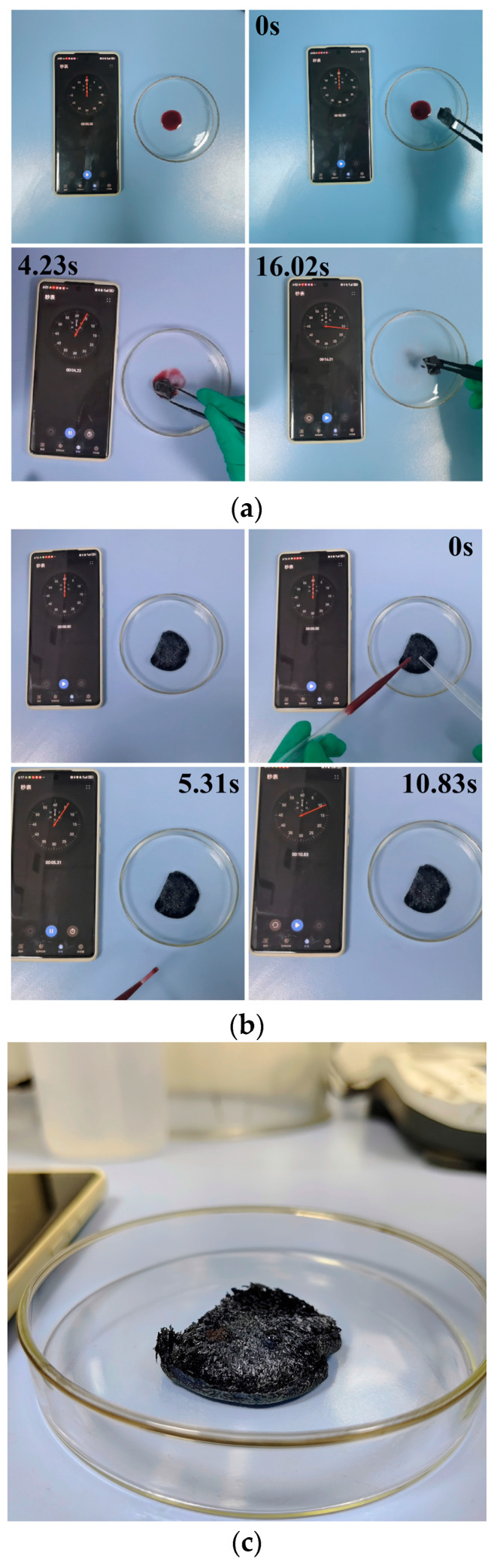
(**a**) Adsorption of M5C10 composite aerogel on soybean oil; (**b**) Adsorption of M5C10 composite aerogel on soybean oil drops and water drops; (**c**) Appearance of soybean oil drops and water droplets after 120 s of standstill.

To further demonstrate the oil-water separation capability of the hydrophobic Ti_3_C_2_T_x_/TOCNF composite aerogels, the M5C10 composite aerogel was selected for adsorption performance testing. The experimental results showed that when 0.0145 g of M5C10 was in contact with 1 mL of amaranth-stained soybean oil, the oil was completely adsorbed within 16.01 s (Figure 9a, Video S1). When 0.0139 g of M5C10 was placed in a petri dish, both distilled water and amaranth-stained soybean oil were added. Then, the soybean oil was found to be completely adsorbed in 10.83 s, while the water droplets remained stable on the surface of the aerogel without permeating (Figure 9b, Video S2). Even after 120 s, the water droplets remained on the M5C10 surface (Figure 9c), which clearly demonstrated aerogel’s excellent hydrophobic properties. The hydrophobic Ti_3_C_2_T_x_/TOCNF composite aerogels exhibited a significant selective adsorption capacity for both water and organic substances. This capability can be attributed to their hydrophobic and oleophilic properties, along with a high specific surface area, as indicated by the results.

#### 3.7.3. Recyclability Performance Analysis

**Figure 10:**
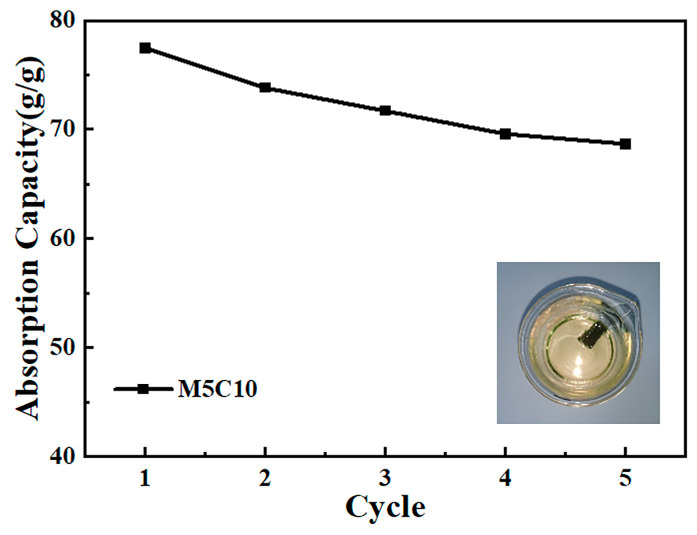
Cyclic adsorption performance of M5C10 composite aerogel on soybean oil.

In the treatment of industrial oily wastewater and wastewater from the catering industry, it is essential for adsorbents to possess a high adsorption capacity and excellent recyclability [37]. Using soybean oil as the target pollutant, the recycling adsorption of hydrophobic Ti_3_C_2_T_x_/TOCNF composite aerogels was investigated. After the M5C10 composite aerogel reached adsorption saturation within 30 min, the adsorbed soybean oil was removed through a desorption experiment for the next adsorption cycle. After five cycles of adsorption experiments, the adsorption capacity of M5C10 gradually decreased from an initial 77.42 g/g to 68.61 g/g, reflecting a consistent reduction rate of approximately 11% (Figure 10). In summary, this indicates that the hydrophobic Ti_3_C_2_T_x_/TOCNF composite aerogels exhibit excellent recyclability, with their structure and performance remaining relatively stable after multiple cycles.

## 4. Conclusions

In this study, a hydrophobic Ti_3_C_2_T_x_/TOCNF composite aerogel exhibiting exceptional oil adsorption performance and reusability was developed. The preparation process involved using TOCNF as a skeleton and Ti_3_C_2_T_x_ as a reinforcing phase, followed by directional freeze-drying. Then, MTCS was utilized as a hydrophobic modifying agent to prepare hydrophobic Ti_3_C_2_T_x_/TOCNF composite aerogels. These aerogels demonstrate excellent hydrophobic properties, effective oil adsorption performance, and reusability.

The water contact angle values of the hydrophobic Ti_3_C_2_T_x_/TOCNF composite aerogels were all above 130°, indicating excellent hydrophobicity. This was due to the chemical reaction that occurred between Ti_3_C_2_T_x_, TOCNF, and MTCS during the chemical vapor deposition process, which effectively removed the original hydrophilic groups. As the concentration of Ti_3_C_2_T_x_ initially increased and then decreased, the oil absorption rate followed a similar pattern, peaking at a Ti_3_C_2_T_x_ mass fraction of 33.3%. The M5C10 composite aerogel demonstrated a high adsorption capacity (with adsorption amounts ranging from 21.5 to 78.2 times its own weight) for four types of organic solvents including ethanol, soybean oil, and vacuum pump oil. M5C10 exhibited particularly outstanding adsorption capacity for dichloromethane, and the maximum adsorption capacity was as high as 98.92 g/g. This capacity surpassed that of several adsorbent materials reported in previous studies. After five cycles of oil adsorption experiments, M5C10 still maintained good adsorption effects, with the reduction rate of the adsorption amount stabilizing at approximately 11%, indicating its excellent recyclability. This research offers valuable insights for addressing environmental pollution issues and establishes a solid foundation for practical applications in the field of oil-water separation, presenting significant prospects for future applications.

## Figures and Tables

**Table 1:** Comparison of Adsorption Amounts of Different Adsorbents on Liquids.

Adsorbent Material	Types of Adsorbed Liquids	Adsorption Capacity (g/g)	References
Graphene oxide@cellulose nanocrystals/EPDM composites	dichloromethane	15.6	[46]
Cellulose-graphene composite aerogels	dichloromethane	36.7	[47]
High-performance hydrophobic aerogel based on nanocellulose, graphene oxide, polyvinyl alcohol, and hexadecyltrimethoxysilane	dichloromethane	24.5	[48]
Superhydrophobic silica aerogel	dichloromethane	17	[49]
Insight into ultra-flexible & robust silica aerogels based on diene synthesis reaction	dichloromethane	15	[50]
Highly compressible and lightweight Al_2_O_3_/PCNF@ANF aerogels	dichloromethane	71.8	[51]
Hydrophobic Ti_3_C_2_T_x_/TOCNF composite aerogel	dichloromethane	98.92	this work

## Data Availability

The original contributions presented in the study are included in the article, further inquiries can be directed to the corresponding author.

## Supplementary Materials

The following supporting information can be downloaded at: https://www.mdpi.com/article/10.3390/polym17030273/s1, Video S1: Adsorption of M5C10 composite aerogel on soybean oil. Video S2: Adsorption of M5C10 composite aerogel on soybean oil drops and water drops.
